# Reward Promotes Self-Face Processing: An Event-Related Potential Study

**DOI:** 10.3389/fpsyg.2016.00735

**Published:** 2016-05-19

**Authors:** Youlong Zhan, Jie Chen, Xiao Xiao, Jin Li, Zilu Yang, Wei Fan, Yiping Zhong

**Affiliations:** ^1^Department of Psychology, Hunan Normal UniversityChangsha, China; ^2^Cognition and Human Behavior Key Laboratory of Hunan Province, Hunan Normal UniversityChangsha, China; ^3^College of Chengnan, Hunan First Normal UniversityChangsha, China

**Keywords:** reward, self-relevant processing, self-face advantage, ERP, P3, LPP

## Abstract

The present study adopted a reward-priming paradigm to investigate whether and how monetary reward cues affected self-face processing. Event-related potentials were recorded during judgments of head orientation of target faces (self, friend, and stranger), with performance associated with a monetary reward. The results showed self-faces elicited larger N2 mean amplitudes than other-faces, and mean N2 amplitudes increased after monetary reward as compared with no reward cue. Moreover, an interaction effect between cue type and face type was observed for the P3 component, suggesting that both self-faces and friend-faces elicited larger P3 mean amplitudes than stranger-faces after no reward cue, with no significant difference between self-faces and friend-faces under this condition. However, self-faces elicited larger P3 mean amplitudes than friend-faces when monetary reward cues were provided. Interestingly, the enhancement of reward on friend-faces processing was observed at late positive potentials (LPP; 450–600 ms), suggesting that the LPP difference between friend-faces and stranger-faces was enhanced with monetary reward cues. Thus, we found that the enhancement effect of reward on self-relevant processing occurred at the later stages, but not at the early stage. These findings suggest that the activation of the reward expectations can enhance self-face processing, yielding a robust and sustained modulation over their overlapped brain areas where reward and self-relevant processing mechanisms may operate together.

## Introduction

The evaluation of the motivational significance and self-relevance of incoming stimuli is an important function of our cognitive system (De Greck et al., 2008). It has been suggested that the brain has evolved specific mechanisms to rapidly allocate reward value as well as self-relevance of events, and these allocations shape our behavior by enhancing specific perceptual and cognitive functions, such as selective attention, working memory, and executive control (Pessoa and Engelmann, 2010). Thus, it is often the case that more attentional and cognitive resources are deployed to stimuli related to motivation and the self, such that these stimuli can be processed more elaborately than stimuli of lesser relevance.

Neuroimaging studies have discovered overlap between reward- and self-relevant processing in core reward circuitry regions, including the ventral striatum (VS), ventral tegmental area (VTA), ventromedial prefrontal cortex (VMPFC), and pregenual anterior cingulated cortex (PACC) (Enzi et al., 2009; Ersner-Hershfield et al., 2009a,b; de Greck et al., 2010). For instance, de Greck et al. (2009) found that the processing of self-relevance and reward overlapped in terms of early signal changes but differed in late signal change in all four regions (right and left NACC, VMPFC, and VTA). Sui and Humphreys (2015) also identified a common mechanism between the processing of self-relevance and reward during perceptual matching, and suggested that self-relevance and reward modulated a common subjective value system. In addition, Northoff and Hayes (2011) recently argued that self-relevance processing was neither integrated nor segregated, but was paralleled with reward-related processing.

The neural and behavioral interactions between reward- and self-relevant processing raise an interesting question: does the activation of the reward system influence self-relevant processing? One possibility is that reward system activation can promote and enhance self-relevant processing. However, the processing of reward- and self-relevant stimuli may be competitive: Reward system activation could serve to weaken self-relevant processing. Moreover, reward-related processing may be independent of self-relevant processing, such that the activation of reward system does not appreciably affect self-relevant processing.

Some neuroimaging studies have demonstrated that reward can generate a sustained activation in task-relevant and value-related brain regions (Krebs et al., 2010; Pessoa and Engelmann, 2010; Veling and Aarts, 2010; Zedelius et al., 2014). For example, a monetary reward can: (1) enhance cue-related task preparation during task-switching by activating the left dorsolateral prefrontal cortex (Savine and Braver, 2010), (2) can promote perceptual and executive control by activating the anterior cingulate cortex (Pessoa, 2009; Pessoa and Engelmann, 2010), (3) can improve value computation by activating the ventral temporal cortex (Philiastides et al., 2010), and (4) can enhance selective attention by activating the primary visual cortex (Stǎnişor et al., 2013). Moreover, recent event-related potential (ERP) researchers have explored the temporal courses of reward effects on some perceptual and cognitive processes. For instance, Hickey et al. (2010) found that an object characterized by a high reward-associated color induced a larger P1 amplitude, reflecting facilitated perceptual activity. Such a stimulus also elicited a large N2pc, indicating the deployment of attention to its location. Additionally, Capa et al. (2013) used a classic reward-priming paradigm to reveal that a high reward cue enhanced parietal P3, which suggested that more working memory resources were invested during executive control of task-switching. Taken together, these studies have suggested that such motivation-driven mechanisms of reward could derive from activation of the task-related neural network. Therefore, we can reasonably speculate that such mechanisms might also play a similar role in self-relevant processing.

Thus, we aimed to use ERPs to determine whether there is an effect of reward on self-face processing, and how such an effect might come about. Given that one’s own face captures very important personal significance in everyday life, the present study employed the faces of the participants as the self-relevant stimuli, and the faces of their friends and stranger as non-self-relevant stimuli. We adopted an implicit self-face recognition paradigm, in which participants were asked to judge the head orientation of faces (right or left; Platek et al., 2006; Devue and Brédart, 2011; Zahavi and Roepstorff, 2011; Yun et al., 2014). Self-face recognition is a commonly used self-processing experimental paradigm, based on the process of distinguishing one’s own face from that of others (Northoff et al., 2006). People typically show faster recognition or enhanced processing of self-face stimuli relative to other-face stimuli, which reflects the specificity of self-face processing (Sui et al., 2006; Sui et al., 2013; Guan et al., 2015).

Event-related potentials studies have shown that the N170 at the occipital-temporal sites or the vertex positive potential (VPP) at the fronto-central sites did not reflect the self-face advantage, but did seem to reflect structural encoding of the face (Bentin and Deouell, 2000; Eimer, 2000; Sui et al., 2006). However, larger N2 amplitudes were elicited by self-faces than by other-faces, which reflected the autonomic and fast recruitment of attentional resources toward self-faces (Keyes and Brady, 2010). Furthermore, larger P3 or late positive potentials (LPP) were also elicited by self-faces compared with other-faces, which showed top-down controlled attentional processing and the cognitive evaluation of self-relevant information (Keyes et al., 2010; Tacikowski and Nowicka, 2010; Devue and Brédart, 2011; Zahavi and Roepstorff, 2011; Guan et al., 2014; Yun et al., 2014). In addition, the P3 and LPP components were also thought to be sensitive to social context (e.g., emotional valence, threat information). Negative faces can elicit larger P3 and LPP amplitudes than positive faces (Guan et al., 2015), and self-concept threat can weaken the self-face advantage by suppressing the processing of self-faces (Guan et al., 2012). Therefore, if reward really can affect self-relevant processing, such an effect is most likely to be observed at the later P3 or LPP stage, but not on the early N2 stage.

Previous studies suggested that both self-relevant and reward-related processing activated some overlapped neural regions (Enzi et al., 2009; Ersner-Hershfield et al., 2009a,b; de Greck et al., 2010) and could share certain mechanisms (Northoff and Hayes, 2011; Sui and Humphreys, 2015). Based on these studies, we sought to clarify their relationship by examining the following hypotheses: (1) that reward can promote and enhance self-relevant processing; (2) that reward could weaken self-relevant processing; or (3) that reward-related processing may be independent of self-relevant processing. It seems reasonable to speculate that if self-relevant processing is enhanced after monetary reward cues (as compared to no reward cue), the first hypothesis would be confirmed. If self-relevant processing is weakened after monetary reward cues, the second hypothesis would be confirmed. Finally, if self-relevant processing is not significantly different between cue conditions, the third hypothesis would be confirmed. Moreover, if the enchantment effect of reward on self-relevant processing really exist, such an effect is most likely to be observed at the late controlled stage indicated by enhanced P3 amplitudes.

## Materials and Methods

### Participants

Eighteen young healthy college students (9 pairs of friends; 8 males; and 10 females; average age was 22.12 years) participated in this experiment. All participants were right-handed, had normal or corrected-to-normal vision, and had no history of neurological or psychiatric disorders. After the experiment, researchers paid the participants, including a basic payment and a task reward, which was later exchanged to money according to the ratio 1000:1 Yuan. Prior to testing, each participant signed an informed consent form. The experiment was approved by the departmental ethics committee.

### Stimuli

According to the standard reward-priming paradigm, coins were often used as a monetary reward cue stimulus (Hickey et al., 2010; Capa et al., 2013). Thus, the front of the ¥100 ($15.40) banknote was used as the reward cue stimulus for these Chinese participants, and the same size blank paper was used as the control stimulus (“no reward cue”; 470 pixels × 220 pixels; Yang et al., 2013). Twenty students completed a 7-point rating scale item (“How much do you desire to get the cue stimuli?”: 1 = no desire at all, 7 = strongly desire) to assess attraction to the ¥100 reward and the same size blank paper. The results showed that the attraction of the 100 Yuan RMB (6.45) was significantly stronger than that toward the same size blank paper [1.22, *t*_(19)_ = 8.34, *p* < 0.001].

The face stimuli consisted of self-face, friend-face, and stranger-face categories, with 20 of each type. Each participant was video recorded (Canon EOS 600D) under studio lighting while assuming a neutral expression and while articulating different speech sounds, according to Chinese vowels and consonants (e.g., ‘ā’, ‘ō’, ‘
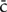
’, ‘
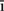
’, ‘ū’, ‘ü’, ‘p’, ‘k’, ‘t’, ‘q’), with the head facing either left or right of 45° (Ma and Han, 2010; Sui et al., 2013). The face images (250 pixels × 250 pixels) consisted of 10 left and 10 right profiles of each face. All faces were shown in gray scale with a neutral facial expression. The mean values of the luminance and contrast of self-faces, friend-faces and stranger-faces were first calculated for each participant. Then, the luminance and contrast of each image were adjusted to the mean value so that they were the same across the three face types. In addition, self-faces were mirror-reversed using Photoshop software, and it was verified that participants were not familiar with the stranger’s face prior to the experiment.

All stimuli were presented on a black background of a 17-inch monitor using E-Prime 2.0 software. Participants were seated in a dim room, at a viewing distance of 75 cm, with the horizontal and vertical visual angles below 5°.

### Experimental Procedure

All participants were asked to perform a head orientation judgment task (left/right), with the face images (i.e., self, friend, or stranger; Platek et al., 2006; Devue and Brédart, 2011; Zahavi and Roepstorff, 2011; Yun et al., 2014) presented after the cue stimuli (e.g., monetary reward or blank paper). Before the formal experiment, participants were instructed that they cannot move their heads when they respond and during the whole experiment, and that if they responded correctly and before 1000 ms to each trial, they would receive the reward presented at the beginning of the trial. The cumulative earnings were displayed at the end of each trial. The participants were informed that the cue stimuli were going to be either a ¥100 banknote or blank paper.

**FIGURE 1:**
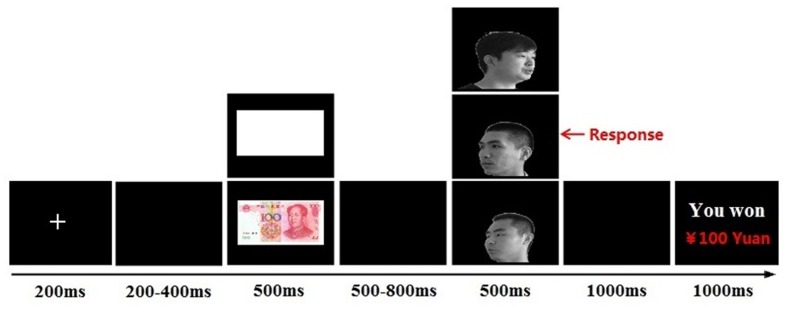
**Single-trial settings.** Each trial started with a fixation (200 ms), followed by a blank screen (200–400 ms, randomly). The cue stimuli (monetary reward/blank paper) was presented for 500 ms. After a blank screen (500–800 ms, randomly), a target face (self-face/friend-face/stranger-face) was presented for 500 ms. After a blank screen for 1000 ms, a feedback about cumulative earnings were presented for 1000 ms.

Each trial started with a fixation cross presented for 200 ms, followed by a blank screen with a variable duration (200–400 ms) and the cue stimulus (¥100 banknote or blank paper; 500 ms). After a blank screen with a randomly variable duration (500–800 ms), a face image (self-face, friend-face, or stranger-face) was presented for 500 ms. The task was to identify the head orientation of faces by pressing the left or right button on a response pad, using the left or right index finger. The participants were instructed to respond as quickly and accurately as possible. After a blank screen for 1000 ms, cumulative earnings were presented for 1000 ms (see **Figure 1**). The experimental session consisted of 360 trials and was divided into two blocks, with a 2-min interval between blocks.

### Electroencephalography (EEG) Recordings and Analysis

Electroencephalograms (EEGs) were continuously recorded using 64 scalp silver/silver- chloride electrodes located in accord with the international 10–20 system, with a ground electrode on the medial frontal aspect. All electrodes were referenced to an electrode at the left mastoid on-line and re-referenced off-line to another electrode at the bilateral mastoid (Petten et al., 2005). Grand averages were calculated after re-referencing individual ERPs to the average mastoid reference. The horizontal electrooculograms (EOGs) were recorded in a bipolar manner from two electrodes placed 1.5 cm lateral to the left and right outer canthi, and the vertical EOG was recorded from electrodes below and above the left eye. The impedance for each electrode was kept below 5 kΩ. EEG was amplified (half-amplitude band pass 0.05–70 Hz) and digitized at a sampling rate of 500 Hz. Off-line trials contaminated by blinks or other artifacts (exceeding ± 50 μv relative to baseline) were corrected using a dipole approach, and EEG activity was referenced to an average. The ERPs epoch in each stimulus condition started 200 ms prior to and ended 800 ms after the target stimulus onset. EEG and EOG activity were processed with a band-pass filter of 0.01–40 Hz, 24 dB/oct, and were average time-locked to target stimulus onset.

**FIGURE 2:**
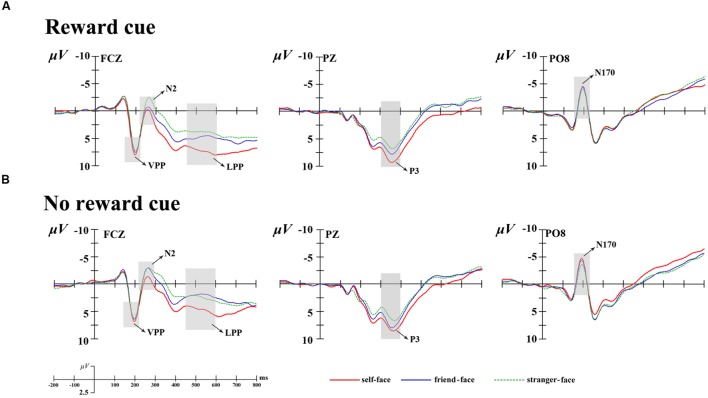
**Averaged event-related potentials (ERPs) at Fcz, Pz, and Po8 for self-face, friend-face and stranger-face in reward cue **(A)** and no reward cue **(B)** condition**.

Combining relevant researches (Platek et al., 2006; Devue and Brédart, 2011; Zahavi and Roepstorff, 2011; Yun et al., 2014) and the visual observation for the brain topography of the grant average ERPs, prominent N1 (100–150 ms), N170 (150–220 ms), VPP (150–220 ms), N2 (220–300 ms), P3 (300–400 ms), and LPP (450–600 ms) components were elicited (see **Figure 2**). All components were measured and analyzed at their corresponding time intervals. Except N170 and VPP components, other componets were measured and analyzed by selecting the following 15 electrode sites for statistical analysis: F3, Fz, F4, FC3, FCz, FC4, C3, Cz, C4, CP3, CPz, CP4, P3, Pz, and P4. A four-way repeated measures analysis of variance (ANOVA) was performed on these measured mean amplitudes for each component. ANOVA factors were cue type (two levels: monetary reward cue and no reward cue), face type (three levels: self-face, friend-face, and stranger-face), laterality (three levels: left, middle, and right sites), and caudality (five levels: frontal, frontocentral, central, centroparietal, and parietal). In additional, the N170 was a special and typical potential to face and was strong at the occipital-temporal sites, which manifested as the VPP at the fronto-central sites (Bentin and Deouell, 2000; Eimer, 2000; Sui et al., 2006; Keyes et al., 2010). Therefore, the N170 was measured and analyzed at the occipital-temporal elecrode sites (PO7, P7, PO8, and P8) and the VPP was measured and analyzed at the fronto-central elecrode sites (F3, Fz, F4, FC3, FCz, and FC4). Their mean amplitudes were coded for condition with cue type (two levels: monetary reward cue and no reward cue) and face type (three levels: self-face, friend-face, and stranger-face), for laterality with two levels (left and right) for the N170 and with three levels (left, middle, and right) for the VPP. The ERP data were analyzed using Brain Products Analyzer software, and the statistical analysis was conducted using SPSS 20.0. Degrees of freedom of the *F*-ratio were corrected according the Greenhouse-Geisser method.

## Results

### Behavioral Results

An ANOVA conducted for Accuracy showed a significant main effect of cue type, *F*_(1,17)_ = 43.66, *p* < 0.001, $\eta_{p}^{2}$ = 0.72, suggesting more accurate recognition of faces after monetary reward (94.10%) than no reward cue (86.30%). However, the main effect of face type and its interaction with cue type were not significant (*p* > 0.05 each). In addition, the ANOVA for RTs showed a significant main effect of cue type, *F*_(1,17)_ = 37.06, *p* < 0.001, $\eta_{p}^{2}$ = 0.69, suggesting faster responses to faces after monetary reward (379.97 ms) than no reward cue (393.79 ms). Again, the main effect of face type and its interaction with cue type were not significant (*p* > 0.05 each).

### ERPs Results

#### N1

**FIGURE 3:**
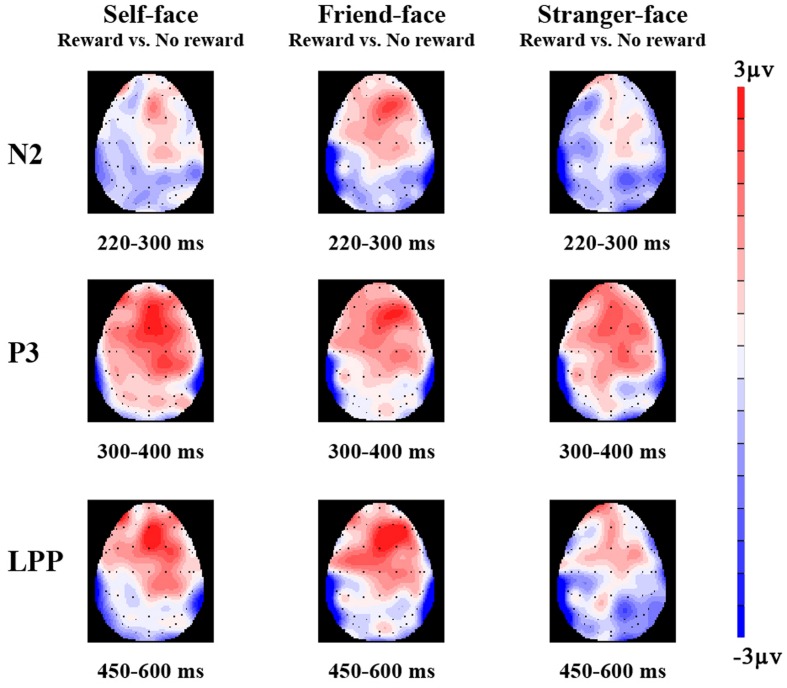
**The topographical maps of voltage amplitudes for reward minus to no reward condition difference ERPs at N2 (220–300 ms), P3 (300–400 ms), and LPP (450–600 ms) of self-face, friend-face, and stranger-face**.

The ANOVA for mean N1 amplitudes showed a main effect of laterality [*F*_(2,34)_ = 24.02, *p* < 0.001, $\eta_{p}^{2}$ = 0.59] and caudality [*F*_(4,68)_ = 8.12, *p* < 0.01, $\eta_{p}^{2}$ = 0.32] (see **Figures 2** and **3**). *Post hoc* comparisons with Bonferroni correction showed that the scalp left lateralized regions (-1.35 μV) showed smaller N1 waves than the middle regions (-0.25 μV, *p* < 0.01) and right lateralized (1.08 μV, *p* < 0.001) regions. The N1 amplitudes were largest at the parietal (0.93 μV) regions and smallest at frontal (-0.95 μV) regions. In addition, the main effect for face type [*F*_(2,34)_ = 0.49, *p* > 0.1, $\eta_{p}^{2}$ = 0.03] and its interaction with cue type did not reach significance [*F*_(2,34)_ = 0.12, *p* > 0.1, $\eta_{p}^{2}$ = 0.01].

#### N170

The ANOVA for mean N170 amplitudes also showed a main effect of laterality, *F*_(2,34)_ = 3.74, *p* < 0.05, $\eta_{p}^{2}$ = 0.18 (see **Figures 2** and **3**), suggesting that the middle regions (0.01 μV) showed more positive N1 waves than the left (-1.41 μV, *p* < 0.05) and right lateralized (-1.45 μV, *p* < 0.05) regions. In addition, none of the main effects for cue type [*F*_(1,17)_ = 0.03, *p* > 0.1, $\eta_{p}^{2}$ = 0.01], face type [*F*_(2,34)_ = 1.15, *p* > 0.1, $\eta_{p}^{2}$ = 0.06] or their interaction [*F*_(2,34)_ = 0.31, *p* > 0.1, $\eta_{p}^{2}$ = 0.00] reached significance.

#### VPP

The ANOVA for mean VPP amplitudes showed a significant interaction between cue type and laterality, *F*_(2,34)_ = 3.36, *p* < 0.05, $\eta_{p}^{2}$ = 0.17 (see **Figures 2** and **3**). There was a significant cue type difference at the scalp middle regions [*F*_(1,17)_ = 3.56, *p* < 0.05], indicating that there were larger VPP mean amplitudes after no reward cue (2.74 μV) than after monetary reward cues (2.24 μV); and whereas no significant difference between cue type at the scalp left [*F*_(1,17)_ = 0.01, *p* > 0.9] and right [*F*_(1,17)_ = 1.02, *p* > 0.3] regions. In addition, no other effect was found (*p* > 0.11 each).

#### N2

The ANOVA for mean N2 amplitudes revealed a marginally significant four-way interactions of cue type × face type × laterality × caudality, *F*_(16,304)_ = 2.83, *p* = 0.08, $\eta_{p}^{2}$ = 0.14. In the frontal regions, there was the main effects of cue type [*F*_(1,17)_ = 4.31, *p* < 0.05, $\eta_{p}^{2}$ = 0.20] and face type [*F*_(2,34)_ = 5.22, *p* < 0.05, $\eta_{p}^{2}$ = 0.24]. There were more negative N2 waves after no reward cue (1.74 μV) than after monetary reward cues (2.04 μV). *Post hoc* comparisons showed that friend-faces (1.74 μV, *p* < 0.05) and stranger-faces (1.48 μV, *p* < 0.05) elicited more negative N2 waves than self-faces (2.45 μV), and whereas no significant difference between friend-faces and stranger-faces (*p* > 0.05). In both the frontocentral and middle regions, there were the main effects of face type [*F*_(2,34)_ = 5.45, *p* < 0.05, $\eta_{p}^{2}$ = 0.24; *F*_(2,34)_ = 4.21, *p* < 0.05, $\eta_{p}^{2}$ = 0.20] and the interaction between cue type and laterality [*F*_(2,34)_ = 3.82, *p* < 0.05, $\eta_{p}^{2}$ = 0.18; *F*_(2,34)_ = 5.84, *p* < 0.01, $\eta_{p}^{2}$ = 0.26], suggesting that the significant differences between cue type were only observed at the scalp middle regions [*F*_(1,17)_ = 9.35, *p* < 0.01; *F*_(1,17)_ = 8.37, *p* < 0.01]. In both the centroparietal and parietal regions, no other effect was found (*p* > 0.14 each).

#### P3

**FIGURE 4:**
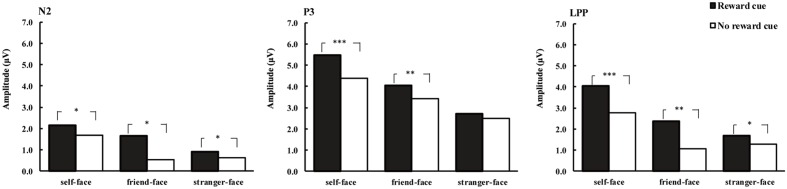
**Average amplitude of N2 (220–300 ms), P3 (300–400 ms), and LPP (450–600 ms) for self-faces, friend-faces and stranger-faces in reward cue and no reward cue condition.** ^∗^*p* < 0.05, ^∗∗^*p* < 0.01, ^∗∗∗^*p* < 0.001.

The ANOVA for mean P3 amplitude demonstrated significant main effects of cue type [*F*_(1,17)_ = 7.69, *p* < 0.05, $\eta_{p}^{2}$ = 0.31] and face type [*F*_(2,34)_ = 7.36, *p* < 0.01, $\eta_{p}^{2}$ = 0.33; **Figures 2**–**4**]. There were larger P3 mean amplitudes after monetary reward (3.97 μV) than no reward cue (2.93 μV). Self-faces elicited larger P3 mean amplitudes (5.48 μV) than friend-faces (4.86 μV, *p* < 0.05) and stranger-faces (3.15 μV, *p* < 0.01), and friend-faces elicited larger P3 mean amplitudes than stranger-faces (*p* < 0.05). The three-way interactions of cue type × face type × laterality [*F*_(4,68)_ = 1.59, *p* > 0.05, $\eta_{p}^{2}$ = 0.08] and cue type × face type × caudality [*F*_(8,136)_ = 1.05, *p* > 0.05, $\eta_{p}^{2}$ = 0.06] were not significant. However, the interaction between cue type and face type was significant, *F*_(2,34)_ = 5.43, *p* < 0.05, $\eta_{p}^{2}$ = 0.26. There was a significant difference for face type after no reward cue [*F*_(1,17)_ = 6.22, *p* < 0.01]; both self-faces (4.37 μV) and friend-faces (3.42 μV) elicited larger P3 mean amplitudes than stranger-faces (2.50 μV), whereas there was no significant difference between self-faces and friend-faces (*p* > 0.05). Moreover, the P3 difference increased after monetary reward cues [*F*_(1,17)_ = 11.03, *p* < 0.001]; self-faces (5.48 μV) elicited larger P3 mean amplitudes than friend-faces (4.03 μV) and stranger-faces (2.72 μV), and friend-faces elicited larger P3 mean amplitudes than stranger-faces (*p* < 0.05).

#### LPP

The ANOVA for mean LPP amplitude demonstrated significant main effects of cue type [*F*_(1,17)_ = 20.14, *p* < 0.001, $\eta_{p}^{2}$ = 0.54] and face type [*F*_(2,34)_ = 16.82, *p* < 0.001, $\eta_{p}^{2}$ = 0.50; **Figures 2**–**4**]. There were larger LPP mean amplitudes after monetary reward (2.68 μV) than no reward cue (1.70 μV). Self-faces elicited larger LPP mean amplitudes (3.39 μV) than friend-faces (1.71 μV, *p* < 0.001) and stranger-faces (1.48 μV, *p* < 0.001), whereas no significant difference between friend-faces and stranger-faces emerged (*p* > 0.05). The three way interactions of cue type × face type × laterality [*F*_(4,68)_ = 0.80, *p* > 0.05, $\eta_{p}^{2}$ = 0.05] and cue type × face type × caudality [*F*_(8,136)_ = 0.53, *p* > 0.05, $\eta_{p}^{2}$ = 0.03] were not significant. However, the interaction between cue type and face type was significant, *F*_(2,34)_ = 3.27, *p* < 0.05, $\eta_{p}^{2}$ = 0.16. There was a significant difference for face type after no reward cue [*F*_(1,17)_ = 13.16, *p* < 0.001]; self-faces (2.76 μV) elicited larger LPP mean amplitudes than both friend-faces (1.07 μV) and stranger-faces (1.28 μV), whereas there was no significant difference between friend-faces and stranger-faces (*p* > 0.05). This LPP difference increased after monetary reward cues [*F*_(1,17)_ = 14.18, *p* < 0.001]; self-faces (4.03 μV) elicited larger LPP mean amplitudes than both friend-faces (2.35 μV) and stranger-faces (1.67 μV), and friend-faces elicited larger LPP mean amplitudes than stranger-faces (*p* < 0.05).

## Discussion

The present study used ERP measures to explore whether and how monetary reward influences self-face processing, seeking to determine whether the influence occurs at the early autonomic attention stage, at a later stage of controlled attention, or both. The findings showed a clear self-relevance effect, such that self-faces elicited larger N2 mean amplitudes than friend-faces and stranger-faces, in both monetary and no reward contexts. However, a cue type by face type interaction effect was observed for both P3 and LPP (450–600 ms) components. Both P3 and LPP differences between self-faces and other-faces were larger in the monetary than the no reward context, and the LPP difference between friend-faces and stranger-faces was also enhanced by reward. These findings demonstrated that the enhancement effect of reward on self-face processing occurred at later P3 and LPP stages, but not at the early N2 stage.

The N1 component was not modulated by face familiarity, consistent with previous reports (Bentin and Deouell, 2000; Eimer, 2000). This component represents the early visual encoding of stimuli (Vogel and Luck, 2000; Luck, 2005). In the present study, no N1 mean amplitude differences were observed between self-faces and other-faces, presumably because the face stimuli are similar in size and luminance for Chinese people. As successful completion of our task required close attention in all conditions, and as image processing and self-other pairings ensured that the stimuli were very similar across conditions, we did not expect and did not find modulation of mean N1 amplitude.

The occipital-temporal N170 and the fronto-central VPP have been proposed to reflect an early stage of ‘structural encoding’ and are not thought to be modulated by face familiarity (Bentin and Deouell, 2000; Eimer, 2000; Sui et al., 2006). Previous ERP studies have also found that such structural encoding of faces reflects automatic processing and was relatively immune to external factors (e.g., attention, threat information, and cultural priming) during the early stage of self-face processing (Sui and Zhu, 2005; Marini et al., 2011; Guan et al., 2012; Sui et al., 2013). Consistent with these studies, we suggest that the structural encoding of faces should not be modulated by their motivational significance. Thus, we inferred that the early stage of self-face processing was possibly immune to the short-term modulation of reward.

The N2 component is considered a neural index of automatic attention responses to highly motivational and salient stimuli, such that larger N2 amplitudes reflect enhanced recruitment of attentional resources (Munro et al., 2007; Schevernels et al., 2014). Previous ERP studies have also found that individuals tend to easily direct their attention to motivationally significant stimuli such as reward and self-relevant stimuli, demonstrating enhanced N2 mean amplitude during processing of these significant stimuli (Hajcak et al., 2010; Krebs et al., 2010; Hughes et al., 2013; Schevernels et al., 2014). Consistent with these findings, the present study found that self-faces elicited larger N2 mean amplitudes than other-faces. The occurrence of our own faces in everyday life may indicate that some significant events (such as criticism, praise, or a warning) will happen to us (Tacikowski et al., 2013). In addition, the present study found that faces associated with reward elicited larger N2 mean amplitudes. At first glance, increased amplitudes in response to non-rewarded faces may suggest increased difficulty deploying automatic attention, probably due to an attentional inhibition process for faces that have to be ignored in order to maximize the subsequent outcome. That could represent an active cognitive process, such as the ability to suppress undesired memory formation (Mecklinger et al., 2009). In contrast, faces that convey motivational significance are able to preferentially engage attention (Adcock et al., 2006). In this respect, it has been suggested that reward might promote the “fine-tuning” of attention, leading to preferential processing of specific events (Engelmann et al., 2009; Pessoa and Engelmann, 2010).

Moreover, we found that participants demonstrated larger P3 mean amplitudes for self-faces than other-faces in the no reward context, and that the size of this self-relevance effect was larger in the monetary reward context. It has been widely reported that the P3 component can be modulated by self-relevance (Keyes and Brady, 2010; Tacikowski and Nowicka, 2010; Fan et al., 2011, 2013; Guan et al., 2014). P3 is related to multiple cognitive funcitions, including top-down controlled attentional processes (Yeung and Sanfey, 2004; Hajcak et al., 2007) as well as cognitive and motivational evaluation (Polich, 2007; Hajcak et al., 2010). Thus, our findings regarding the self-relevance effect could be explained by the fact that self-faces garner a larger amount of attentional and cognitive resources and thus evoke enhanced motivational responses than other-faces. Previous studies have also found that the processing of faces associated with reward could be related to the encoding of value-prediction codes in which motivational evauation come into play (Yeung and Sanfey, 2004; Yeung et al., 2005; Mennes et al., 2008; Kamarajan et al., 2009). Thus, when a paticular face is known to potentially produce a reward, these value-prediciton codes allow the enhanced allocation of attentional and cognitive resources to distinguish self-faces and other-faces.

Furthermore, the enhancement of reward on the self-relevance effect was still observed for the LPP (450–600 ms), and such a difference between friend-faces and stranger-faces was also promoted by reward. Recent studies have suggested that the P3 component may reflect the initial allocation of attention to motivationally salient stimuli, whereas the later LPP may reflect selective attention for high self-relevant stimuli, and thus be more specifically related to stimulus significance (Schupp et al., 2006; Foti and Hajcak, 2008; Hajcak et al., 2009). Northoff et al. (2009) found that self-relevant stimuli often contained very strong and important emotional meaning and value. Thus, self-relevance may play a pivotal role in the selection of the input stimulus for further processing self-faces in reward context. In addition, previous workers have reported that close relationships can result in self-other overlap, which was explained in terms of shared cognitive and neural representations of the self and close others (such as a mother or close friends) in collectivistic individuals (Aron et al., 1991; Zhu et al., 2007; Wang et al., 2012; Myers et al., 2014; Tan et al., 2015). Moreover, Aron et al. (2013) believed that these individuals were inclined to process information about close others in a similar way to how they process information about themselves. Thus, participants processed friend-faces in a similar way to processing self-faces, explaining why the enhancement of reward on friend-faces was also larger than toward stranger-faces at the LPP component.

These findings suggest that reward can promote and enhance self-relevant processing at the later controlled attention stages, but not at the early automatic attention stage. Moreover, the activation of reward expectations could yield robust and sustained modulation over their overlapped brain areas where reward and self-relevant processing mechanisms may operate together (Enzi et al., 2009; Ersner-Hershfield et al., 2009a,b; de Greck et al., 2010). Thus, we suspect that the reward circuits were activated by monetary stimuli, and then this activation was working to drive or potentiate the neural networks associated with self-relevant processing, which could then enhance neural responses for self-relevant stimuli.

In additional, it was also worth noted that the experimental task of the present study was to response to the head’s orientation immediately after the face onset, thus the ERPs to faces might be contaminated by response preparation and execution. In order to control this potential contamination, in the study of Sui et al. (2013), ERPs were measured to non-target faces (own or the friend’s faces), and participants were not required to make response to these faces (Sui et al., 2013). However, no significant effects involving response were observed in their study, thus they collapsed the ERPs across the target and non-target respond conditions. Future studies, using the experimental task of head orientation judgment, should realize the potential contamination and control this contamination by modifying the experimental design.

## Conclusion

Taken together, in addition to the enhancement effect of reward on perceptual and cognitive processing such as task-switching, executing control, and perceptual matching reported in the previous studies, the present study further showed that reward can enhance social cognitive processing, such as that associated with self-relevance. This enhancement effect occurred at the late stage of top-down controlled processing, as indexed by the P3 and LPP (450–600 ms) components, but not at the early automatic attention stage, as indexed by the N2 component. These findings suggest that reward can influence self-perception in an implicit manner. Future studies should adopt other incentive stimuli (such as social reward) and self-relevant stimuli to investigate the enhancement effect of reward on self-processing, particularly using high-spatial-resolution fMRI to uncover neural substrates that mediate this enhancement effect, and how these neural activities are related to the pursuit for reward.

## Author Contributions

YZ, JC, XX, WF, and YZ designed the experiments. YZ, JL, and ZY recruited participants and collected the data. YZ and XX performed the data analyses. YZ and JC wrote the manuscript.

## Conflict of Interest Statement

The authors declare that the research was conducted in the absence of any commercial or financial relationships that could be construed as a potential conflict of interest.
